# Study of Self-Locking Structure Based on Surface Microstructure of Dung Beetle Leg Joint

**DOI:** 10.3390/biomimetics9100622

**Published:** 2024-10-14

**Authors:** Dexin Sun, Sen Lin, Yubo Wang, Jiandong Cui, Zhiwei Tuo, Zhaohua Lin, Yunhong Liang, Luquan Ren

**Affiliations:** 1 College of Mechatronics, Changchun Polytechnic, Changchun 130033, China; sundexin2007aa@163.com; 2 School of Mechanical and Aerospace Engineering, Jilin University, Changchun 130025, China; linsen23@mails.jlu.edu.cn (S.L.); linzhaohua@jlu.edu.cn (Z.L.); 3 The Key Laboratory of Bionic Engineering, Ministry of Education, Jilin University, Changchun 130025, China; wyb24@mails.jlu.edu.cn (Y.W.); cuijd22@mails.jlu.edu.cn (J.C.); liangyunhong@jlu.edu.cn (Y.L.); lqren@jlu.edu.cn (L.R.); 4 State Key Laboratory of Material Processing and Die & Mould Technology, School of Materials Science and Engineering, Huazhong University of Science and Technology, Wuhan 430074, China; 5 Institute of Structured and Architected Materials, Liaoning Academy of Materials, Shenyang 110167, China

**Keywords:** dung beetle, surface microstructure, self-locking structure, folding mechanism, bionic design

## Abstract

Dung beetle leg joints exhibit a remarkable capacity to support substantial loads, which is a capability significantly influenced by their surface microstructure. The exploration of biomimetic designs inspired by the surface microstructure of these joints holds potential for the development of efficient self-locking structures. However, there is a notable absence of research focused on the surface microstructure of dung beetle leg joints. In this study, we investigated the structural characteristics of the surface microstructures present in dung beetle leg joints, identifying the presence of fish-scale-like, brush-like, and spike-like microstructures on the tibia and femur. Utilizing these surface microstructural characteristics, we designed a self-locking structure that successfully demonstrated functionality in both the rotational direction of the structure and self-locking in the reverse direction. At a temperature of 20 °C, the biomimetic closure featuring a self-locking mechanism was capable of generating a self-locking force of 18 N. The bionic intelligent joint, characterized by its unique surface microstructure, presents significant potential applications in aerospace and various engineering domains, particularly as a critical component in folding mechanisms. This research offers innovative design concepts for folding mechanisms, such as those utilized in satellite solar panels and solar panels for asteroid probes.

## 1. Introduction

Over the course of billions of years of evolution, organisms have developed a variety of unique and efficient biological structures that exhibit remarkable mechanical properties [1,2,3,4,5]. Many of these structures are characterized by multistage designs, which include specialized joint macrostructures, fibrous layer microstructures, and surface microstructures. Notably, the leg joints of dung beetles demonstrate an exceptional capacity for load bearing, enabling them to push objects significantly heavier than their own body weight, garnering considerable interest due to their superior mechanical properties [6]. Nevertheless, traditional artificial joints continue to face numerous challenges related to load-bearing capacity, durability, and self-locking capabilities. As advancements in aerospace and other fields progress, there is an increasing demand for functional diversification and high performance in folding mechanisms with the self-locking function emerging as a particularly pressing issue. Aerospace spacecraft, for instance, frequently require self-locking mechanisms to facilitate the expansion and retraction of various equipment components. Consequently, investigating the microstructure of dung beetle leg joints and developing a self-locking structure inspired by these biological models has become a significant and challenging area of research.

In recent years, the fields of biomechanics and bionic engineering have experienced significant advancements, leading to an intensified investigation into the biological structures found in nature. Researchers both domestically and internationally have conducted comprehensive studies on the microstructure, strength distribution, and geometric topology of various biomaterials, including the elytra and flight wings of beetles and other insects, yielding noteworthy findings [7,8,9,10,11,12,13,14,15]. The examination of elytra has yielded significant insights for the development of high-performance cavity and locking structures. In recent years, researchers both domestically and internationally have investigated beetle leg joints as a natural rotational mechanism, noting their attributes of rotatability, high strength, toughness, wear resistance, and self-cleaning properties. Konstantin Nadein [16] found a protein-based lubricating substance in the femur–tibial joints of *Zophobas morio* beetles to achieve joint wear resistance. Konstantin Nadein [17] also studied the self-cleaning system composed of “hair”, “brush” and “scraper” structures in the legs of Pachnoda marginata beetles, which can ensure that there is no debris in the joints. Steffen Vagts [18,19] studied the wear resistance mechanism of leg joints in Pachnoda marginata beetles. The current study reveals that certain leg joints of beetles exhibit self-cleaning and wear-resistant properties, which are attributed to the microstructural characteristics of these joints. The beetle leg is anatomically divided into two segments: the tibia and the femur. The interaction of the microstructures between these segments during rotation serves as a mechanism for the self-cleaning function. Specifically, the microstructural features at the joint interface between the tibia and femur reduce the contact area between the two segments. The presence of distinct curvature, height, and microstructural elements contributes to a minimized actual contact area, which may also facilitate a self-locking mechanism. Furthermore, the design of insect leg joints that minimizes friction can be achieved through specific geometrical configurations at various length scales. While the majority of existing research has concentrated on self-cleaning and friction resistance, there is a notable scarcity of studies that explore the application of the microstructural attributes of beetle leg joints in the design of self-locking structures. Meanwhile, the leg joints of dung beetles, as a typical representative of high-load-bearing parts, have a high research value [6,20,21,22,23,24]. It is necessary to study the microstructure and bionic design of dung beetle leg joints. Bionic structure design can provide many design ideas and inspiration for the engineering field [21,25,26,27,28,29].

In this study, the morphology, size and distribution of the surface microstructure of dung beetle leg joints were studied in detail. There are three kinds of microscopic raised structures on the surface of the tibia, which are fish scale, brush and spike-like structures, while there is only one brush structure on the surface of the femur. A self-locking structure based on fish scale and brush structures is designed, and its self-locking function is tested. On this basis, the self-locking structure is applied to the bionic joint, and the intelligent bionic closure can generate 18 N of self-locking force at 20 °C. A series of scenarios are designed to apply a bionic joint with a self-locking structure to the folding mechanisms of aerospace spacecraft to demonstrate its rotatable and self-locking functions. This design provides a new idea and method for the design of key components such as mechanical arms and aerospace folding mechanisms.

## 2. Materials and Methods

### 2.1. Specimen Handling and Sample Preparation

Dung beetles were purchased from insect stores on the internet. In the lab, dung beetles were fed in beetle feeding boxes. Before the experiment, the dung beetles were first placed at 5 °C to enter a dormant state and then placed at −10 °C for euthanasia.

### 2.2. Scanning Electron Microscopy

We wiped the surface of the sample with anhydrous ethanol to remove impurities and then sprayed a layer of gold powder with a thickness of 10 μm on the surface of the sample with an auto sputter coater to improve the electrical conductivity of the sample to ensure the scanning electron microscope image effect.

### 2.3. Micro-Computed Tomography

We imported the model into Dragonfly, set the 3D preset to “Plastic”, color table to “rainbow”, and opacity to “Double door slope”, and obtained a clear 3D model. Then, we created a new ROI in the image segmentation section, selected the brush in the 2D view tool to color the beetle leg in the three slice plots, and applied the difference value to X after all the colors were applied to obtain the 3D reconstruction model.

### 2.4. Preparation of Bionic Sample with Self-Locking Microstructure

Firstly, the friction test samples of three microstructures were drawn by 3DMax software (3Dmax2018) and then formed by Formlabs Form4 3D printing (Guangzhou Shape You Technology Co., Ltd., Guangzhou, China). We put the sample in the box and poured silica gel. After curing for 24 h, we removed the sample to the mold. Then, the epoxy resin, polycaprolactone and low melting point alloy were mixed at a weight ratio of 20:1:180 and stirred in a water bath at 80 °C at a speed of 500 rpm for 30 min; then, we added 5 g of curing agent and continued to stir in a water bath at 80 °C at a speed of 500 rpm for 5 min. Then, it was vacuumed at 80 °C for 3 min for treatment. Finally, the mixture was poured into the prepared mold, solidified at 50 °C for 8 h, and removed from the mold, and the bionic microstructure sample for friction testing was obtained.

### 2.5. Friction Testing

When two kinds of microstructure plate parts to be tested were held by a tension clamp, the friction in the direction of the inverse structure was defined as the two structures being close to each other, and the friction in the direction of the structure was defined as the two structures being far from each other. In the separation test along the structure, the two structures changed from coincidence to separation; in the inverse structural coincidence test, the two structures went from separate to coincidence. The heat gun was used in the heating state, and the temperature was measured by a contact thermometer.

### 2.6. Material Preparation of Bionic Joint

Preparation method of epoxy resin: The mass ratio of epoxy resin E51 and 650 polyamide curing agent was mixed according to the ratio of 10:5. The mixture was magnetically stirred in a water bath at 80 °C at 500 rpm for 5 min; then, the mixture was placed in a vacuum drying oven for vacuum at 80 °C for 3 min, after which the mixture was poured into a homemade mold. Finally, the mixture was cured for 8 h in a blast drying oven at 50 °C and then removed from the mold. Preparation method of composite: The composite was prepared by mixing epoxy resin, polycaprolactone and a low melting point alloy at a weight ratio of 20:1:180, and then we stirred the mixture in a heated water bath at 80 °C at 500 rpm for 30 min. Then, we added 5 g curing agent, continued to stir the mixture in a water bath at 80 °C at 500 rpm for 5 min, and then vacuumed at 80 °C for 3 min for defoaming. Finally, the mixture was poured into the mold, cured at 50 °C for 8 h, demolded and removed.

### 2.7. Composition of Bionic Joint

The composite blocks were placed in the mold at an angle, and then the mold was filled with epoxy resin (15 × 5 × 1 mm). After curing, it was taken out as a fiber layer, and the angle between the two adjacent layers was 90°. The four layers were stacked and put into the mold (90 × 10 × 5 mm), and we continued to add epoxy resin to fill it. After curing, the bionic exoskeleton was obtained. The bionic exoskeleton had the same gradient hardness structure from the outside to the inside (epoxy resin on the outside, composite material on the inside) as the leg exoskeleton of dung beetles, and the cross-layered structure of fiber blocks was the same as that of dung beetles. We 3D printed joints with microstructures and then used silicone to make the inverted mold, and then we used the above method to prepare the bionic exoskeleton to prepare the side wall to obtain the bionic joint.

### 2.8. Application of Bionic Joint

The application of the bionic joint was carried out on a satellite model (Taobao China Software Co., Ltd., Hangzhou, China), which was purchased from the network. The femur of the bionic joint was prepared by mold, and the tibia was prepared by 3D printing. The femur and tibia segments were attached to the satellite solar panels and then connected together. A string attached to the tibia was connected to a shape-memory polymer or other drive device, and the solar panel could be unfurled by pulling the string tight.

### 2.9. Statistical Analysis

All data and error calculations were analyzed by Excel (Office 2021) and Origin (Origin 2021). The dimensions of the joint microstructure and biomimetic microstructure of dung beetle legs were obtained by scanning the contour curve, and multiple microstructures were scanned simultaneously as parallel tests. Similarly, when testing the self-locking properties of microstructures, there are many points of interaction between microstructures on the curves obtained when rotating or sliding microstructures. In short, each peak in the force–displacement curve can be viewed as a parallel test result of the interaction between the microstructures.

## 3. Results and Discussion

### 3.1. Microstructure and Morphology of the Leg Joint of Dung Beetle

**Figure 1:**
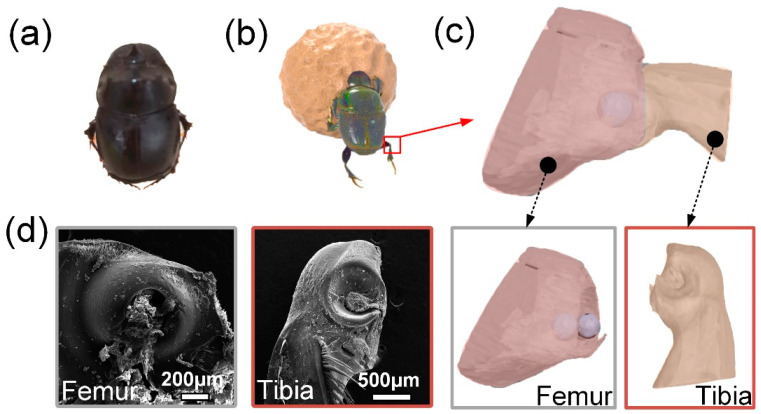
(**a**) Overall morphology of dung beetles; (**b**) a dung beetle pushing a ball; (**c**) three-dimensional reconstruction model of dung beetle leg joint; (**d**) SEM images of dung beetle leg joints.

Figure 1a,b illustrate the morphology and behavioral patterns of dung beetles, which primarily consume animal feces and exhibit the ability to form fecal balls. During the process of transporting these dung balls, the leg joints of dung beetles are subjected to significant forces over extended periods, suggesting that these joints possess exceptional mechanical properties. Among the various factors contributing to the enhanced mechanical performance of dung beetle leg joints, structural characteristics are particularly influential. Therefore, ZEISS Xradia 610 Versa scanner Micro-CT scanning (Carl Zeiss AG, Shanghai, China) was performed on the dung beetle leg joint first, followed by 3D modeling, and then the SEM images were compared to verify whether the 3D reconstruction model was reasonable. Figure 1c presents a three-dimensional reconstruction model of the leg joint of a dung beetle, comprising the tibia and femur. The distal end of the tibia features a depression shaped like two hemispheres, while the proximal end of the femur exhibits a corresponding bulge of two hemispheres. The SEM image depicted in Figure 1d clearly illustrates the structural details on the lateral aspects of both the tibia and femur. Additionally, microstructural features are observed on the surfaces of the joint components, which may contribute to enhanced stability and load-bearing capacity by increasing the contact area, providing additional friction and a locking mechanism. In order to understand the surface microstructure, size and other parameters of the joint, the surface of the leg joint was further tested by XL-30 ESEM SEM (Thermo Fisher Technology China Co., Ltd., Shanghai, China).

**Figure 2:**
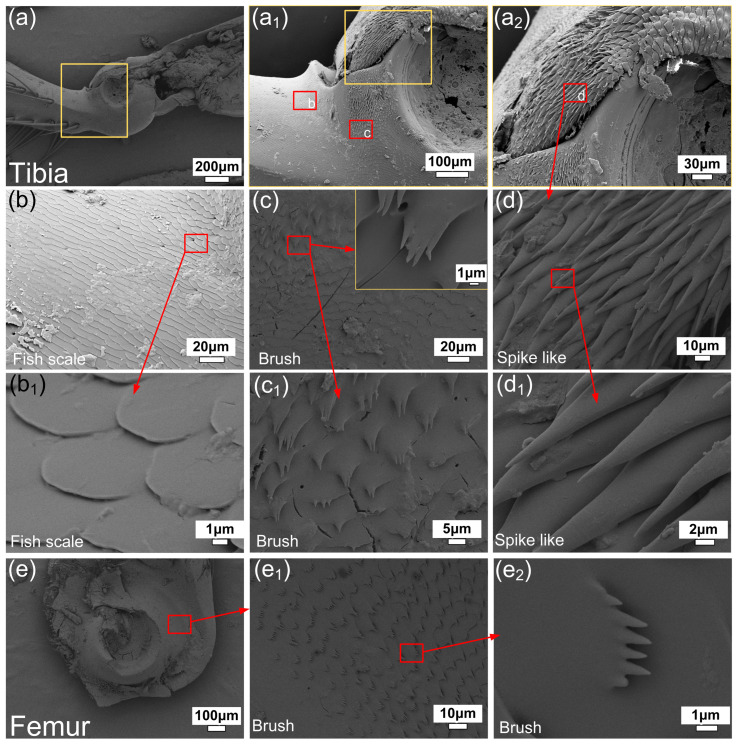
SEM images of microstructures at the surface joints of dung beetle leg joints. (**a**,**a_1_**,**a_2_**) Tibia and its surface microstructure; (**b**,**b_1_**) fish-scale-like microstructure; (**c**,**c_1_**) brush-like microstructure; (**d**,**d_1_**) spike-like microstructure; (**e**,**e_1_**,**e_2_**) femur and its surface microstructure.

The leg joint of the dung beetle was separated, and SEM images were taken on the surface of the tibia and femur, respectively, to study the surface morphology of the joint of the dung beetle leg. Studies have shown that the microstructure of beetle joints is similar, which mainly plays a role in anti-pollution, lubrication and anti-wear [16,17,18,19,20,21]. As can be seen from Figure 2a–d, there are three kinds of micro-convex structures on the surface of tibia, namely, fish scale, brush and spike like, which exist in different parts of the tibia, respectively. The femur surface in Figure 2e has only one brush-like structure. The fish-scale structure on tibia is outside the joint and does not come into direct contact with the femur surface. The scaly microstructures gradually change into stepped microstructures from the periphery, which is similar to the transition of adjacent brush-like microstructures. The single fish scale is similar to a circle with a radius of 3 μm, and the fish-scale microstructure mainly plays the role of anti-fouling and secreting joint lubricants [16,17,18,19,20,21]. The surface of the tibia in the joint is bristle, and the direction of the bristle microstructure is consistent with the direction of the tibia rotating around the leg. The single brush-like microstructure is about 5 μm long, and the ends are divided into one to six smaller spikes. The femur surface in the leg joint is similar to the tibia surface and also has a brush-like microstructure. The individual length is shorter about 3 μm, and the terminal spikes are more about 1 to 7 μm. The contact surface of the tibia and femur inside dung beetle leg joints is a brush-like microstructure that touches itself, which plays a role of wear resistance and anti-fouling. The brush can be rotated to brush out dirt, while the brush microstructure can reduce the contact surface during friction. The bottom of the tibia hemispherical depression is a spiny microstructure, and the direction of the spiny microstructure is consistent with the rotation direction of the tibia around the leg segment. The length of a single spiny microstructure is about 25 μm. Longer spike-like structures can further drain contaminants from the leg joints.

**Figure 3:**
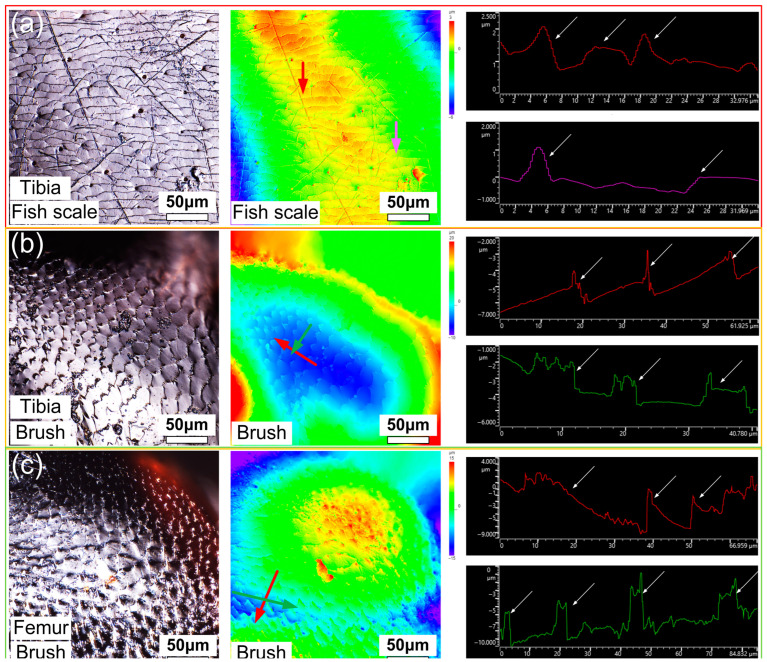
(**a**) Fish-scale microstructure on the surface of tibia segment; (**b**) brush-like microstructure on the surface of the tibia segment; (**c**) laser confocal image, height cloud map, and some microstructural contour lines of the surface microstructure on the surface of the femur segment with a brush-like microstructure. The color arrow in the height cloud image is the position of extracting the contour line, and the color of the arrow matches the color of the final contour line.

The surface microstructure of the tibia and femur was further characterized by laser confocal microscopy. In Figure 3a,b, the same fish scale and brush-like microstructure as in SEM images can be seen. The curvature of the surface where the spike-like structure is located is large, and it is difficult to photograph the spike-like structure with a light mirror. The large height difference in the cloud image is caused by the curved surfaces of the tibia and femur exoskeletons. Here, a section with the same height is selected to extract contours to represent the geometry of the microstructure. The colored arrows in the height cloud map represent the length and direction of the contours taken. The height of the fish-scale microstructure on the surface of tibia is about 0.4~1.2 μm, and the spacing between the two adjacent layers is about 6~12 μm. The height of the brush structure on the surface of the tibia is about 2~3 μm. The spacing between two adjacent microstructures perpendicular to the brush direction is about 9~12 μm, and the spacing between two adjacent microstructures parallel to the brush direction is about 15~20 μm. The height of the brush structure on the surface of the femur is about 4~8 μm, the spacing between two adjacent microstructures perpendicular to the brush direction is about 20~40 μm, and the spacing between two adjacent microstructures parallel to the brush direction is about 10~20 μm. The surface microstructure of the leg joint mainly provides anti-fouling and wear-resisting functions during leg joint rotation. In addition to providing high wear resistance to the leg joints of dung beetles, joint secretions are also present. Holes secreting lubricating liquid can be seen in Figure 2b,c and Figure 3a,b. The microstructure plays an auxiliary role in improving the wear resistance, and it will produce a self-locking phenomenon under certain conditions [16,17,18,19,20,21]. At this point, we obtained the surface structure and size parameters of the dung beetle foot joint, and then based on this, we designed the bionic microstructure, realized the self-locking function through the bionic microstructure coordination, and then applied it in the bionic joint. The information obtained here on the surface microstructure size and morphology of dung beetle leg joints can be used to inspire the design of bionic joints, which has great inspiration for engineering design. However, for biological research, the study sample is too small to support any conclusion of biological relevance. 

### 3.2. Bionic Self-Locking Structure Design and Performance Research

**Figure 4:**
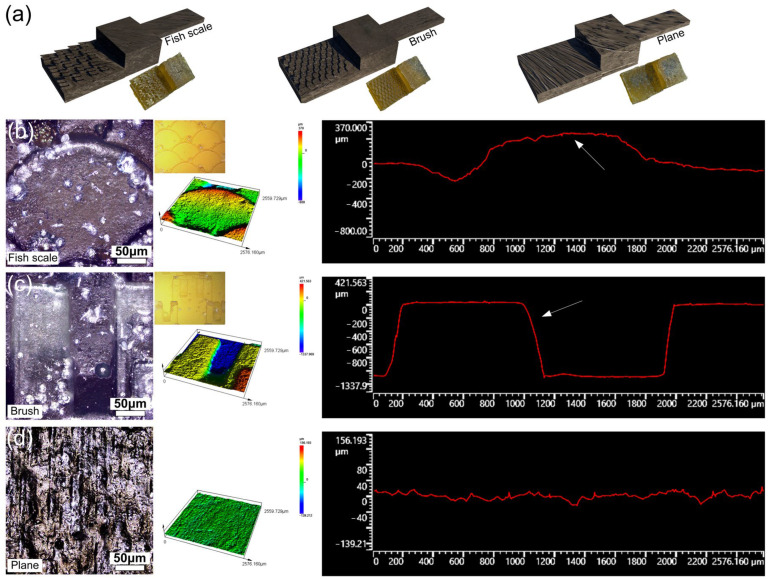
(**a**) Schematic and physical drawings of microstructure samples with fish scale, brush and plane; (**b**) fish scale, (**c**) brush, and (**d**) plane photomicrography, laser confocal, and contour curves of the microstructure. The white arrow indicates a microstructural unit.

Bionic microstructures were designed and prepared according to the size of microstructures on the tibia and femur of dung beetle leg joints and the spacing between adjacent microstructures. The surface microstructure of dung beetle leg joints is not exactly the same, and only the typical characteristic structure is referred to for a simplified bionic design. The fish-scale structure comprising microstructural features of cross-stacking semi-circular sheet structures is shown in Figure 2b. The brush structure features of the bifurcation on the main body are shown in Figure 2e. In this way, the fish-scale structure is smooth, the brush structure has many edges and corners, and the two cooperate with each other to ensure better mechanical properties in the case of self-locking. The most important thing is that the smooth edges of the fish-scale structure can prevent jamming, and the sharp edges of the brush structure can ensure self-locking. The bionic self-locking structure and size are determined in accordance with the purpose of easy preparation by mold casting technology. All in all, the bionic self-locking structure and size are optimized based on the typical characteristics of the surface microstructure of the dung beetle leg joint and prepared by mold casting technology. As shown in Figure 4a, three structures, the fish scale, brush and plane, were designed according to the microstructures on the surface of dung beetle leg joints studied in the previous paper. It is hoped that these microstructures can be combined to achieve a self-locking function. The combination of fish scale and brush can realize the self-locking function, and the plane structure is used for friction testing. According to the research, the microstructure of the leg joint of beetles may have self-locking function [19], and the microstructure is prepared on the bionic joint designed in the previous research, hoping to increase the self-locking function of the bionic joint [6]. The fish-scale surface consists of about a quarter of a cylinder, and the brush-like structure is a single cuboid with ends divided into three small cuboids. Figure 4b,c show the morphology characteristics and size information of the three surface structures. The individual fish-scale structures are about 300 μm in height and 1200 μm in width. The height of a single brush structure is about 1050 μm, the length is 5000 μm, and the width is 800 μm. The distance between two adjacent small brushes is 1000 μm. The height difference of the planar structure is about 10 μm, which is used to test the frictional properties of the fish-scale structure and the brush structure on the plane.

**Figure 5:**
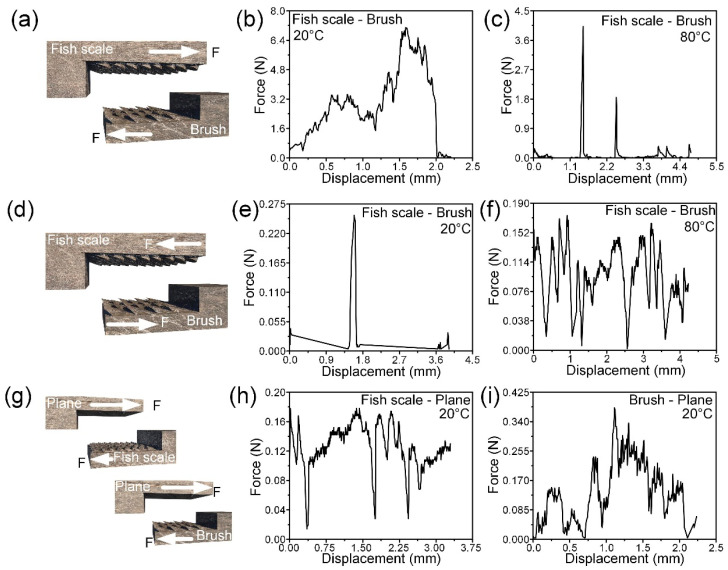
(**a**) The force direction diagram of the fish-scale structure and the brush-like structure; force–displacement curves of fish-scale and brush-like structures against structural friction at (**b**) 20 °C and (**c**) 80 °C; (**d**) the force direction diagram of the fish-scale structure and the brush-like structure; force–displacement curves of fish-scale and brush-like structures at (**e**) 20 °C and (**f**) 80 °C; (**g**) the force direction diagram of the fish-scale structure and plane, brush structure and plane; (**h**) force–displacement curve of the friction of the fish-scale structure and plane at 20 °C; (**i**) force–displacement curve of brush structure and plane friction at 20 °C.

Figure 5a–i show the mechanical properties of bionic self-locking structures. The proximity of two structures to each other is defined as the inverse structural direction friction, and the distance between two structures is defined as the prostructural direction friction. The shape memory material used in the previous study was used to prepare the sample [6,30] by comparing the self-locking function between different structures at different temperatures. The shape memory epoxy resin has a high strength at room temperature, which can effectively ensure the self-locking function, and a low strength at high temperature, similar to rubber, which can ensure effective rotation. As shown in Figure 5a–c, the fish scale and brush-like structures rub against the direction of the structure at 20 °C, and the maximum self-locking force is about 7.2 N and 4.1 N at 80 °C. As shown in Figure 5d–f, the fish scale and brush-like structures rub along the structure direction at 20 °C, and the maximum self-locking force is about 0.264 N, while it is about 0.171 N at 80 °C. As shown in Figure 5g–i, at 20 °C, the maximum friction force generated by the fish scale and flat structure is about 0.185 N, and the maximum friction force generated by the brush and flat structure is about 0.395 N. It can be seen that the force–displacement curve obtained during the test is zigzag, which is formed by the interaction of the two parts of the microstructure. Each increase and decrease in force can be regarded as an interaction between the microstructures, and a force–displacement curve contains the results of multiple tests. It can be seen from the test results that the fish-scale structure and brush structure cooperate with each other to have a good self-locking ability in the direction of the reverse structure, while the movement along the direction of the structure has almost no force, and heating can also reduce the self-locking force. The above experimental results prove that the bionic self-locking structure can realize single-direction self-locking. The self-locking function of the bionic self-locking structure is provided by both the structure and material. The bionic structure can rotate in the forward direction and reverse direction, and the material can unlock after self-locking, which makes the bionic self-locking structure more functional. Different functions can be obtained by preparing bionic self-locking structures with different materials, which improves the application potential of bionic joints. The most typical example is the use of metal materials such as TC4 to make bionic self-locking structures, which can obtain unlockable self-locking structures. Using polymers with different glass transition temperature (T_g_) to prepare bionic self-locking structures can realize unlocking at different temperatures. For example, polymer materials with higher T_g_ (T_g_ > 200 °C) need to be selected for the space environment to prevent failure. For daily environmental use, polymer materials with lower T_g_ (T_g_ < 100 °C) are usually selected to facilitate triggering and unlocking.

### 3.3. Application of Bionic Self-Locking Structure in Bionic Joint

**Figure 6:**
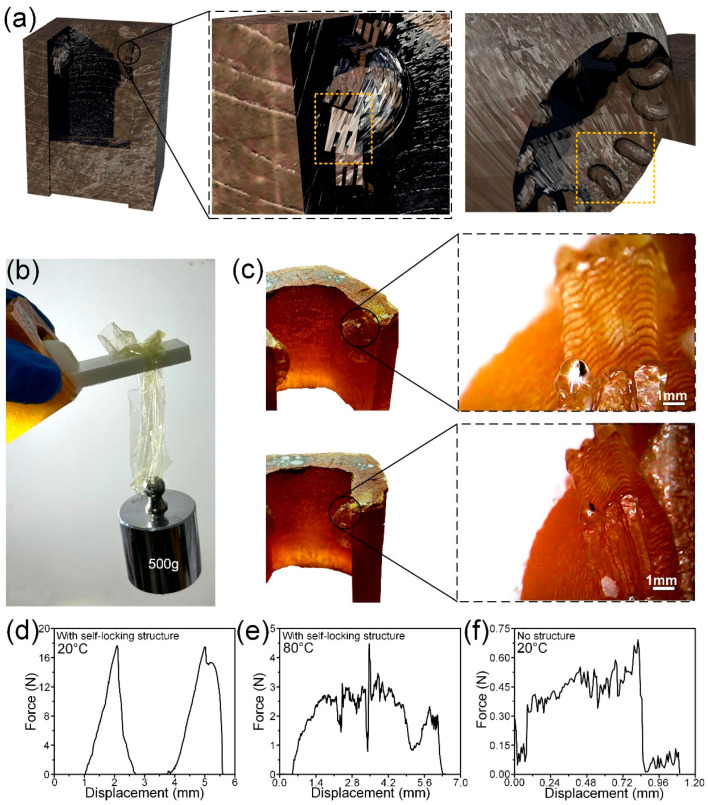
(**a**) Schematic diagram of a bionic joint with a self-locking structure. The orange dotted lines are self-locking microstructures; (**b**) bionic joint with self-locking structure to withstand 500 g weights; (**c**) bionic joints with self-locking structures; (**d**) self-locking force of bionic joint with self-locking structure at 20 °C; (**e**) self-locking force of bionic joint with self-locking structure at 80 °C; (**f**) the friction force of a structurally free bionic joint when rotated at 20 °C.

Figure 6a–c shows a bionic intelligent joint with a self-locking structure and its self-locking performance. According to the distribution of microstructures on the surface of dung beetle leg joints, the brush-like microstructures were prepared at the joints of the legs. The femur part of the bionic joint is fabricated by the mold, and the tibia part is prepared by polymer 3D printing. To facilitate the installation of tibia and femur, the interstitial space is larger than the dung beetle leg joint, so the angle of the scaly structure of the tibial portion is changed to 90° perpendicular to the tibial surface. It can be seen in Figure 6b that the bionic joint can withstand a load of 500 g when it locks itself. In Figure 6c, it can be seen that the microstructures are successfully prepared to the joints of the bionic joints. In Figure 6d–f, it can be seen that the bionic intelligent lock can produce a self-locking force of 18 N at 20 °C and a self-locking force of 4.5 N at 80 °C. The friction force of the joint without the self-locking structure is about 0.68 N during rotation. The experimental results show that the bionic intelligent joint with a microstructure and self-locking function has been successfully prepared. The bionic self-locking structure shows excellent self-locking performance in joints, but the fatigue performance has not been further studied. Cyclic performance is an important property to be considered in the application of self-locking structures, but fatigue performance is more related to the intrinsic properties of materials. The material used in this paper is shape memory resin. High strength rotating at low temperatures will be directly destroyed; low strength at high temperatures similar to rubber materials can withstand long-term repeated loads. At the same time, the self-locking structure is intended to be used in the scene of one-time self-locking such as the satellite folding mechanism, so the fatigue performance of the self-locking structure is not discussed in this paper. In the follow-up research, different materials can be used to prepare bionic self-locking structures for fatigue properties. For example, oriented carbon fibers are added to the resin to make composite materials, or thermoplastic polymers are added to induce phase separation to improve their toughness. The fatigue properties of bionic self-locking structures can be improved by modifying materials or replacing high-strength and high-toughness materials.

**Figure 7:**
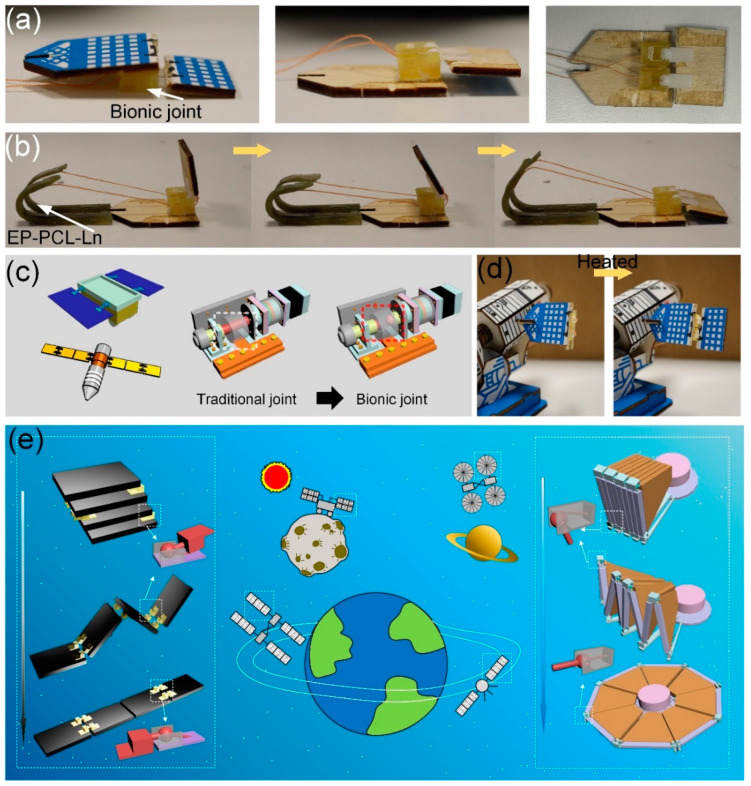
(**a**) Model of a satellite solar panel fitted with a bionic joint; (**b**) driving the satellite solar panel model to unfold and self-lock; (**c**) traditional bearing structure and bionic joint; (**d**) the satellite solar panel model deploys and self-locks; (**e**) the application potential and mechanism design of bionic joints in the aerospace field. The dotted lines and arrows point to bionic joints with self-locking structures.

Bionic intelligent joints that incorporate shape memory materials with superior mechanical properties and self-locking capabilities exhibit significant potential for application in aerospace folding mechanisms. In the aerospace sector, mechanisms such as satellite panels and solar panels are characterized by a one-time deployment, meaning they are typically opened only once and are not designed to be retracted. This characteristic provides an advantageous context for the bionic self-locking structure, which demonstrates exceptional self-locking performance as discussed in this paper. To assess its practical applicability, the aforementioned bionic intelligent joint with a self-locking structure has been integrated into a model of a satellite solar panel for evaluation. As shown in Figure 7a,b, two adjacent solar panels are connected through two bionic joints, one solar panel is fixed on the tibia, and the other is fixed on the femur. The two adjacent solar panels can then rotate around the joint. The driver drives the tibia to rotate and thus drives the solar panel to rotate, which can be easily rotated along the structural direction, but when the solar panel is unrolled, it locks itself and cannot be rotated against the structural direction. As shown in Figure 7c, replacing common bearing joints in engineering with bionic joints can improve strength and bring a self-locking function. Replacing the traditional through-bearing joint with a bionic joint with a self-locking structure can eliminate the motor for self-locking in some cases. There are various folding mechanisms on spacecraft such as lunar lander and manned spacecraft. Figure 7c–e shows the broad application prospects of bionic intelligent joints in the aerospace field. Bionic joints with a self-locking structure can be well used in folding and fan folding mechanisms. In the follow-up research, the fatigue properties of bionic self-locking structures will be further studied to expand its application potential. For example, the bionic self-locking structure applied to folding-screen mobile phones, laptops and other electronic products needs to withstand multiple rotation, self-locking and unlocking, and it requires lightweight materials. Modifying or replacing the base material to improve the overall fatigue performance can make the bionic self-locking structure have the opportunity to be applied to electronic products such as folding-screen mobile phones.

## 4. Conclusions

In this study, the surface microstructure of the dung beetle leg joint was studied, and based on this, the bionic microstructure was designed on the surface of the bionic joint hemispherical joint. The intelligent properties of bionic articulation joints are successfully added, the self-locking of bionic articulation joints is realized, and the application potential of bionic articulation joints is expanded. The surface of the dung beetle leg joint has a fish-scale-like, brush-like and spike-like microstructures that are anti-fouling and wear-resistant. Through the microstructural design of the bionic joint surface of the hemispheres, the function of rotation in the direction of the structure and self-locking in the direction of the reverse structure is realized. At 20 °C, the bionic joint can produce a self-locking force of 18 N, and at 80 °C, the intelligent joint can produce a self-locking force of 4.5 N. The friction force of the bionic joint without a self-locking structure is about 0.68 N. Compared with the bionic joint without a self-locking structure, the bionic intelligent joint can achieve multi-function and intelligence. The bionic intelligent joint with a microstructure on the surface of the joint has a wide application prospect in aerospace and other engineering fields as a key component of the folding mechanism, which provides a new design idea for the folding mechanism such as satellite solar panels and asteroid probe solar panels.

## Data Availability

The original contributions presented in the study are included in the article; further inquiries can be directed to the corresponding author.

## References

[1] Yang R., Zaheri A., Gao W., Hayashi C., Espinosa H.D. (2017). AFM identification of beetle exocuticle: Bouligand structure and nanofiber anisotropic elastic properties. Adv. Funct..

[2] Chen Y., Ma Y., Yin Q., Pan F., Cui C., Zhang Z., Liu B. (2021). Advances in mechanics of hierarchical composite materials. Compos. Sci. Technol..

[3] Wegst U.G., Bai H., Saiz E., Tomsia A.P., Ritchie R.O. (2015). Bioinspired structural materials. Nat. Mater..

[4] Huang W., Shishehbor M., Guarín-Zapata N., Kirchhofer N.D., Li J., Cruz L., Wang T., Bhowmick S., Stauffer D., Manimunda P. (2020). A natural impact-resistant bicontinuous composite nanoparticle coating. Nat. Mater..

[5] Grunenfelder L.K., Milliron G., Herrera S., Gallana I., Yaraghi N., Hughes N., Evans-Lutterodt K., Zavattieri P., Kisailus D. (2018). Ecologically driven ultrastructural and hydrodynamic designs in stomatopod cuticles. Adv. Mater..

[6] Tuo Z., Yang K., Ma S., Cui J., Shi Y., Zhao H., Liang Y., Liu C., Lin Z., Han Z. (2024). Multi-Level Structural Enhancement Mechanism of the Excellent Mechanical Properties of Dung Beetle Leg Joint. Small.

[7] Rivera J., Murata S., Hosseini M.S., Trikanad A.A., James R., Pickle A., Yaraghi N., Matsumoto N., Yang W., Parkinson D.Y. (2021). Structural design variations in beetle elytra. Adv. Funct..

[8] Rivera J., Hosseini M.S., Restrepo D., Murata S., Vasile D., Parkinson D.Y., Barnard H.S., Arakaki A., Zavattieri P., Kisailus D. (2020). Toughening mechanisms of the elytra of the diabolical ironclad beetle. Nature.

[9] Sun J., Bhushan B. (2012). Structure and mechanical properties of beetle wings: A review. RSC Adv..

[10] Scalet J.M., Sprouse P.A., Schroeder J.D., Dittmer N., Kramer K.J., Kanost M.R., Gehrke S.H. (2022). Temporal changes in the physical and mechanical properties of beetle elytra during maturation. Acta Biomater..

[11] Lomakin J., Huber P.A., Eichler C., Arakane Y., Kramer K.J., Beeman R.W., Kanost M.R., Gehrke S.H. (2011). Mechanical properties of the beetle elytron, a biological composite material. Biomacromolecules.

[12] Dai Z., Yang Z. (2010). Macro-/micro-structures of elytra, mechanical properties of the biomaterial and the coupling strength between elytra in beetles. J. Bionic Eng..

[13] He C., Zu Q., Chen J., Noori M.N. (2015). A review of the mechanical properties of beetle elytra and development of the biomimetic honeycomb plates. J. Sandw. Struct. Mater..

[14] Kundanati L., Signetti S., Gupta H.S., Menegon M., Pugno N.M. (2018). Multilayer stag beetle elytra perform better under external loading via non-symmetric bending properties. J. R. Soc. Interface.

[15] Goczał J., Beutel R.G. (2023). Beetle elytra: Evolution, modifications and biological functions. Biol. Lett..

[16] Nadein K., Kovalev A., Thøgersen J., Weidner T., Gorb S. (2021). Insects use lubricants to minimize friction and wear in leg joints. Proc. R. Soc. B Biol. Sci..

[17] Nadein K., Gorb S. (2022). Smart joints: Auto-cleaning mechanism in the legs of beetles. Commun. Biol..

[18] Dai Z., Gorb S.N. (2004). Micro-structure and frictional characteristics of beetle’s joint. Sci. China Phys. Mech..

[19] Nadein K., Gorb S. (2022). Lubrication in the joints of insects (Arthropoda: Insecta). J. Zool..

[20] Vagts S., Schlattmann J., Kovalev A., Gorb S.N. (2020). Structure and frictional properties of the leg joint of the beetle *Pachnoda marginata* (Scarabaeidae, Cetoniinae) as an inspiration for technical joints. Biomimetics.

[21] Vagts S., Schlattmann J., Kovalev A., Gorb S.N. (2018). The topology of the leg joints of the beetle *Pachnoda marginata* (Scarabaeidae, Cetoniinae) and its implication for the tribological properties. Biomimetics.

[22] Sun J.Y., Guo Y.J., Tong J. (2006). Testing methods for nanoindentation property of the cuticle of bovine hoof wall and dung beetle’s foreleg femur. J. Terramechanics.

[23] Zhang Z., Jia H., Sun J., Tong J. (2016). Nanoindentation investigation of the stress exponent for the creep of dung beetle (*Copris ochus* Motschulsky) cuticle. Bioengineered.

[24] Oh J.K., Behmer S.T., Marquess R., Yegin C., Scholar E.A., Akbulut M. (2017). Structural, tribological, and mechanical properties of the hind leg joint of a jumping insect: Using katydids to inform bioinspired lubrication systems. Acta Biomater..

[25] Wang Y., Jiang X., Li X., Ding K., Liu X., Huang B., Ding J., Qu K., Sun W., Xue Z. (2023). Bionic ordered structured hydrogels: Structure types, design strategies, optimization mechanism of mechanical properties and applications. Mater. Horiz..

[26] Li J., Chen Q., Zhang Q., Fan T., Gong L., Ye W., Fan Z., Cao L. (2020). Improving mechanical properties and biocompatibilities by highly oriented long chain branching poly (lactic acid) with bionic surface structures. ACS Appl. Mater. Interfaces.

[27] Gu Y., Yu L., Mou J., Wu D., Zhou P., Xu M. (2020). Mechanical properties and application analysis of spider silk bionic material. e-Polymers.

[28] Sun Z., Gong Y., Bian Z., Zhang J., Zhao L., Hu N. (2024). Mechanical properties of bionic lattice and its hybrid structures based on the microstructural design of pomelo peel. Thin-Walled Struct..

[29] Wang W., Lu L., Lu X., Liang Z., Tang B., Xie Y. (2022). Laser-induced jigsaw-like graphene structure inspired by Oxalis corniculata Linn. leaf. Bio-Des. Manuf..

[30] Tuo Z., Chen K., Zhou Q., Wang Y., Wang Q., Zhang Y., Lin Z., Liang Y. (2024). High-performance shape memory epoxy resin with high strength and toughness: Prepared by introducing hydrogen bonds through polycaprolactone and low melting point alloy. Compos. Sci. Technol..

